# Comparison of clinical efficacy and psychological outcomes of femoral neck system combined with rhBMP-2 for young adult femoral neck fractures

**DOI:** 10.3389/fsurg.2026.1935352

**Published:** 2026-08-28

**Authors:** Dan Li, Wanrong Yang

**Affiliations:** 1 Department of Orthopaedics, Fuyang People’s Hospital Affiliated to Anhui Medical University, Fuyang, Anhui, China; 2 Clinical Research Center for Spinal Deformity of Anhui Province, Fuyang, Anhui, China

**Keywords:** anxiety and depression, femoral neck fracture, femoral neck system, internal fixation, rhBMP-2

## Abstract

**Objective:**

This study aimed to compare postoperative clinical efficacy and psychological status between isolated Femoral Neck System (FNS) fixation and FNS combined with local recombinant human bone morphogenetic protein-2 (rhBMP-2) implantation in patients aged 18–55 years with unstable femoral neck fractures.

**Methods:**

A retrospective clinical analysis was performed on 65 patients aged 18–55 years admitted to our institution from January 1, 2022 to December 1, 2023. All participants were divided into two treatment groups: Group A received FNS internal fixation alone, while Group B underwent FNS fixation combined with intraoperative rhBMP-2 implantation at the fracture gap. Intraoperative indicators including operative duration, fluoroscopy frequency and intraoperative blood loss were recorded. Preoperative and final follow-up Self-Rating Depression Scale (SDS) and Self-Rating Anxiety Scale (SAS) scores were collected for psychological assessment. Long-term postoperative complications, fracture healing progress, femoral neck shortening and bilateral femoral offset differences were also compared between cohorts. Postoperative radiographs were applied to evaluate fracture reduction quality and internal fixation positioning, and the Harris Hip Score (HHS) was adopted to quantify postoperative hip joint function.

**Results:**

The overall follow-up period spanned 6 to 35 months, with an average follow up duration of 25.11 ± 7.35 months. The age range of subjects was 23–53 years, with an upper age limit of 55 years defined as the cut-off for young adult patients in this study. No statistically significant intergroup differences were detected in operative time, intraoperative blood loss or fluoroscopy frequency (*P* > 0.05). In Group A, six patients developed avascular necrosis of the femoral head, three experienced femoral neck shortening, and two suffered fracture nonunion. At the final follow up, significant bilateral discrepancies in femoral neck length and femoral offset remained relative to the uninjured hip (*P* < 0.05). In Group B, only one case of femoral head necrosis, two cases of femoral neck shortening and one case of fracture nonunion were identified, with no statistically significant bilateral differences in femoral neck shortening or femoral offset at the last follow up (*P* > 0.05). Both groups demonstrated obvious postoperative declines in SDS and SAS scores, yet no between group disparity was found in final Harris hip functional scores (*P* > 0.05).

**Conclusions:**

FNS combined with local rhBMP-2 implantation was associated with shorter fracture healing time, lower complication rates, and improved radiographic outcomes compared with FNS alone, while hip function remained comparable between groups. Both groups demonstrated significant postoperative reductions in SDS and SAS scores.

## Introduction

Femoral neck fractures (FNFs) account for approximately 3.58% of all skeletal injuries across all age groups (1). In young adult patients, generally defined as individuals aged 18–60 years according to previous clinical criteria (2), femoral neck fractures are mostly induced by high-energy traumatic forces, producing strong vertical shear stress, steep fracture planes, and compromised mechanical stability. Restricted by the unique fragile blood supply of the femoral neck, the overall fracture union rate remains as low as 9.3%. Clinical evidence indicates that the incidence of severe postoperative complications including fracture nonunion and avascular necrosis of the femoral head ranges from 30% to 60% after internal fixation, making FNFs a persistent clinical challenge for orthopedic surgeons (3).

Young adults bear heavy social and family responsibilities; persistent joint dysfunction or femoral head necrosis after injury will severely impair physical function, mental wellbeing and daily living quality. Therefore, developing optimized surgical protocols for young patients with unstable femoral neck fractures carries prominent clinical significance.

With continuous advances in orthopedic implant technology, multiple fixation modalities have been widely applied to treat femoral neck fractures. The dynamic hip screw (DHS) provides reliable mechanical stability yet requires lengthy surgical incisions, prolonged operation time and extensive soft tissue dissection. Cannulated compression screws (CCS) represent a minimally invasive alternative but offer insufficient anti-shear capacity, forcing patients to maintain prolonged bed rest and delay rehabilitation training. Previous comparative research reported a 46.7% complication rate and 22% reoperation risk for DHS fixation, vs. 35.1% complications and 20% revision risk for CCS fixation (4, 5). To balance minimally invasive manipulation and biomechanical stability, the AO Research Institute developed the Femoral Neck System (FNS), which integrates the minimally invasive implantation advantages of hollow screws and the strong anti-rotation performance of DHS.

Recombinant human bone morphogenetic protein-2 (rhBMP-2) is an osteoinductive growth factor belonging to the transforming growth factor-β superfamily. It promotes mesenchymal stem cell recruitment, proliferation, and osteogenic differentiation, thereby enhancing bone regeneration. rhBMP-2 has been clinically applied in conditions associated with impaired bone healing. Because femoral neck fractures are characterized by compromised local vascularity and limited biological healing potential, local rhBMP-2 augmentation combined with stable fixation may provide complementary mechanical and biological advantages. Although FNS has gained expanding clinical popularity, few clinical studies have explored the combined application of FNS and rhBMP-2 for young unstable femoral neck fractures. In addition, psychological recovery after traumatic fractures has received increasing clinical attention. Patients with femoral neck fractures frequently experience anxiety, depression, and concerns regarding postoperative functional recovery, particularly those of working age who often face substantial family and occupational responsibilities. Nevertheless, previous studies evaluating FNS have primarily focused on radiographic and functional outcomes, while psychological recovery has rarely been investigated as an important component of postoperative prognosis. Moreover, most existing relevant research only focuses on radiographic and functional recovery outcomes while neglecting perioperative psychological fluctuations in fracture patients. This retrospective study compared clinical efficacy and psychological recovery between isolated FNS fixation and FNS combined with local rhBMP-2 implantation, aiming to provide clinical evidence for individualized treatment plans targeting young femoral neck fractures.

## Materials and methods

### General clinical data

This retrospective study enrolled 65 patients diagnosed with femoral neck fractures and treated in the Department of Orthopedics at Fuyang People's Hospital Affiliated to Anhui Medical University between January 1, 2022 and December 1, 2023. All enrolled subjects were assigned to two treatment groups: Group A received FNS internal fixation alone, while Group B received FNS fixation supplemented with intraoperative rhBMP-2 implantation.

According to previous clinical criteria, femoral neck fractures occurring in patients aged 18–60 years are classified as young adult femoral neck fractures (2). Considering the characteristics of our single-center cohort, patients aged 23–53 years were included in this study.

Group A contained 33 patients (19 males and 14 females) aged 24 to 52 years, with a mean age of 36.24 ± 6.90 years. The Garden fracture classification distribution included three type I fractures, nine type II fractures, sixteen type III fractures and five type IV fractures. Group B consisted of 32 patients (18 males and 14 females) aged 23 to 53 years, with a mean age of 39.88 ± 8.37 years, including four type I fractures, seven type II fractures, fifteen type III fractures and six type IV fractures. All patients completed preoperative x-ray and three dimensional CT examinations to confirm fracture diagnosis.

Inclusion criteria: age younger than 55 years; fracture sustained within three weeks of trauma; treatment via either FNS alone or FNS combined with rhBMP-2; complete clinical and long term follow up data available.

Exclusion criteria: pathological fractures; severe systemic osteoporosis; preexisting cognitive dysfunction or mental illness; metabolic disorders contraindicating surgical intervention.

This research obtained formal ethical approval from the hospital's Medical Ethics Committee [Approval No. Medical Ethics Review (2022) No. 31].

### Surgical procedures

#### Group A (FNS monotherapy group)

Patients were placed in a supine position on a fracture traction table, and C-arm fluoroscopy was utilized to verify satisfactory fracture reduction. The surgical area underwent routine sterile disinfection, and the screw entry point was positioned at the lesser trochanter level under fluoroscopic guidance. A 5 cm skin incision was created with layered subcutaneous dissection, and appropriate anteversion angle was maintained to preserve the native neck-shaft angle. A guide pin was inserted through the guide sleeve; anteroposterior fluoroscopy confirmed the pin was positioned in the middle and inferior third of the femoral neck, while lateral fluoroscopy verified central alignment within the femoral neck. The guide pin tract was expanded with a trapezoidal drill, depth measurement was completed, and the FNS femoral neck load bearing rod was implanted. The rod was locked with a locking screw, followed by anti-rotation screw insertion and gradual compression. Final intraoperative fluoroscopy was performed to confirm stable fracture reduction.

#### Group B (FNS combined with rhBMP-2 fixation)

**Figure 1:**
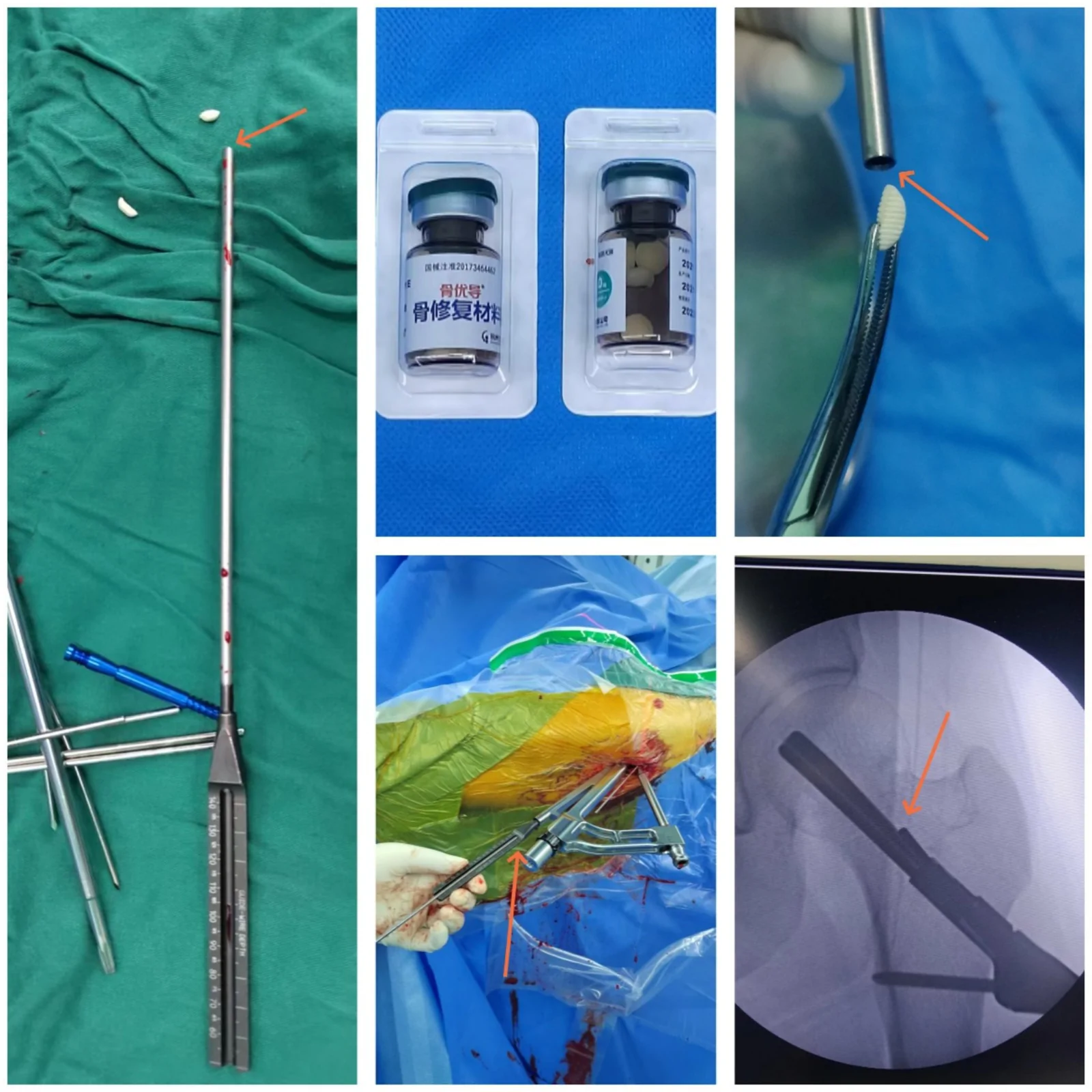
Local implantation of recombinant human bone morphogenetic protein-2 (rhBMP-2) at the femoral neck fracture site. rhBMP-2 was implanted into the fracture site of the femoral neck using a hollow depth gauge during surgery.

The surgical procedure replicated all steps adopted for Group A. After securing the FNS load-bearing rod with a locking screw, rhBMP-2 material was implanted into the fracture gap using a hollow depth gauge (Figure 1). An anti-rotation screw was subsequently inserted and tightened to achieve compression. The wound was closed layer by layer and covered with sterile surgical dressing.

### Postoperative management

All patients received standardized postoperative care: anticoagulant medication administered within 24 h after surgery to prevent deep vein thrombosis; prophylactic antibiotics to reduce surgical site infection risk; and structured rehabilitation training guided by professional physical therapists.

### Observation indicators

**Figure 2:**
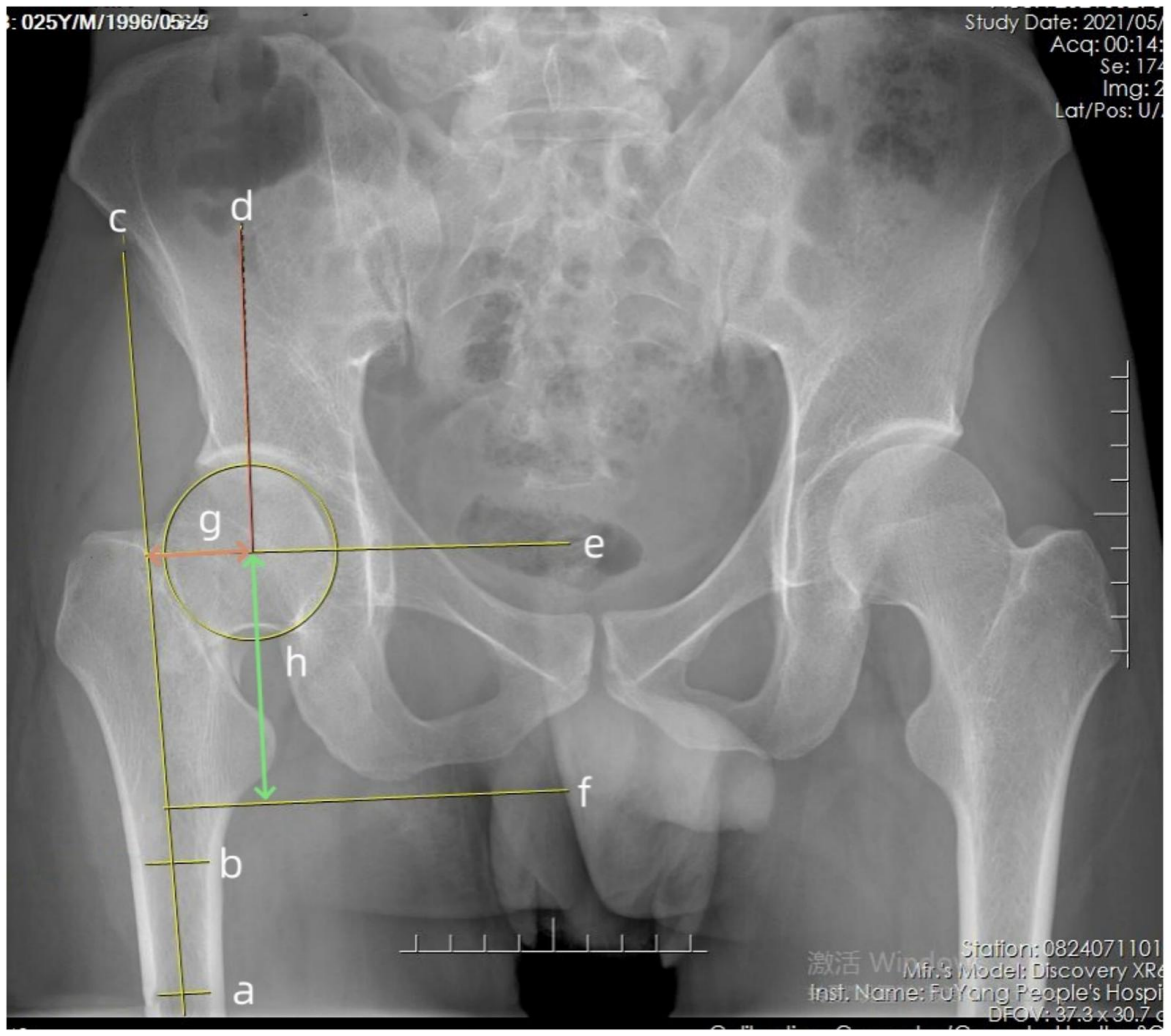
Radiographic measurement methods for femoral neck shortening and femoral offset (FO). Anteroposterior pelvic radiograph demonstrating the measurement of femoral neck shortening and FO. The axis of the femoral shaft (c) was defined by the midpoint of the femoral medullary cavity (a,b). The perpendicular distance from the femoral head center (d) to the femoral shaft axis (c) represented femoral offset (FO). The distance (h) between the center of the femoral head (e) and the upper edge of the lesser trochanter was measured perpendicular to the femoral shaft axis (c) to evaluate femoral neck length. FO, femoral offset.

**Figure 3:**
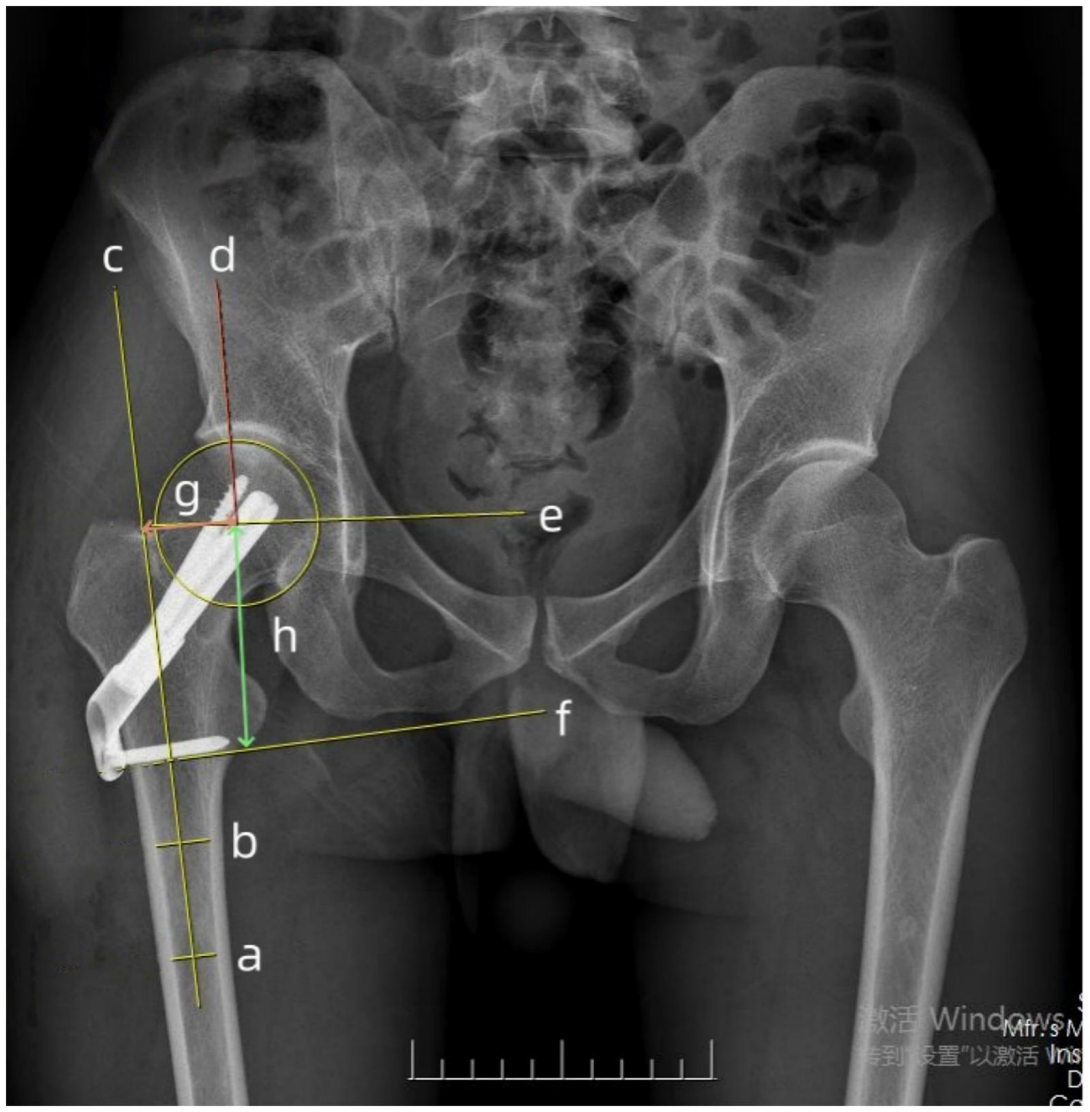
Measurement of femoral neck shortening and femoral offset after femoral neck fracture fixation. Representative radiographic measurements of femoral neck shortening and femoral offset after surgical treatment of femoral neck fractures. FO, femoral offset.

Preoperative and final follow up SDS and SAS scores were recorded to evaluate psychological status in both groups. Additional observational endpoints included operative duration, intraoperative fluoroscopy frequency, total intraoperative blood loss, fracture healing cycle, postoperative bilateral femoral offset variation (Figure 2), and complications such as femoral neck shortening (Figure 3) and avascular necrosis of the femoral head. Complete postoperative weight-bearing was defined as the ability of patients to bear full body weight on the affected limb without walking assistance, as determined by clinical examination and radiographic evidence of satisfactory fracture healing. Fracture healing time was defined as the interval from surgery to the first radiographic evidence of continuous cortical bridging across the fracture site with disappearance of the fracture line, accompanied by the absence of pain during weight-bearing. Routine postoperative radiographs were used to assess fracture reduction quality and internal fixation positioning, while the Harris Hip Score was adopted to evaluate long-term hip joint functional recovery.

### Statistical analysis

All clinical data were independently input into Excel spreadsheets by two trained researchers, and statistical processing was performed using SPSS 26.0 software. Categorical data were analyzed via the *χ*^2^ test; continuous measurement data were expressed as mean ± standard deviation and compared using independent sample *t*-tests. A *P*-value less than 0.05 was defined as the threshold for statistically significant differences.

## Results

### Baseline characteristics

No statistically significant differences were observed between Group A and Group B regarding baseline demographic characteristics, including gender composition, patient age, and Garden fracture classification (*P* > 0.05; Table 1). The two groups were comparable in terms of preoperative characteristics.

**Table 1:** Comparison of general information (gender, age, and fracture classification) between the two groups of patients.

Group	*N*	Age	Sex (male/female)	Fracture classification (Garden classification)			
				I	II	III	IV
A	33	36.24 ± 6.90	19/14	3	9	16	5
B	32	39.88 ± 8.37	18/14	4	7	15	6
*t*/*χ*^2^		−1.912	0.012	0.501			
*p*		0.060	0.914	0.919			

### Operative outcomes

**Figure 4:**
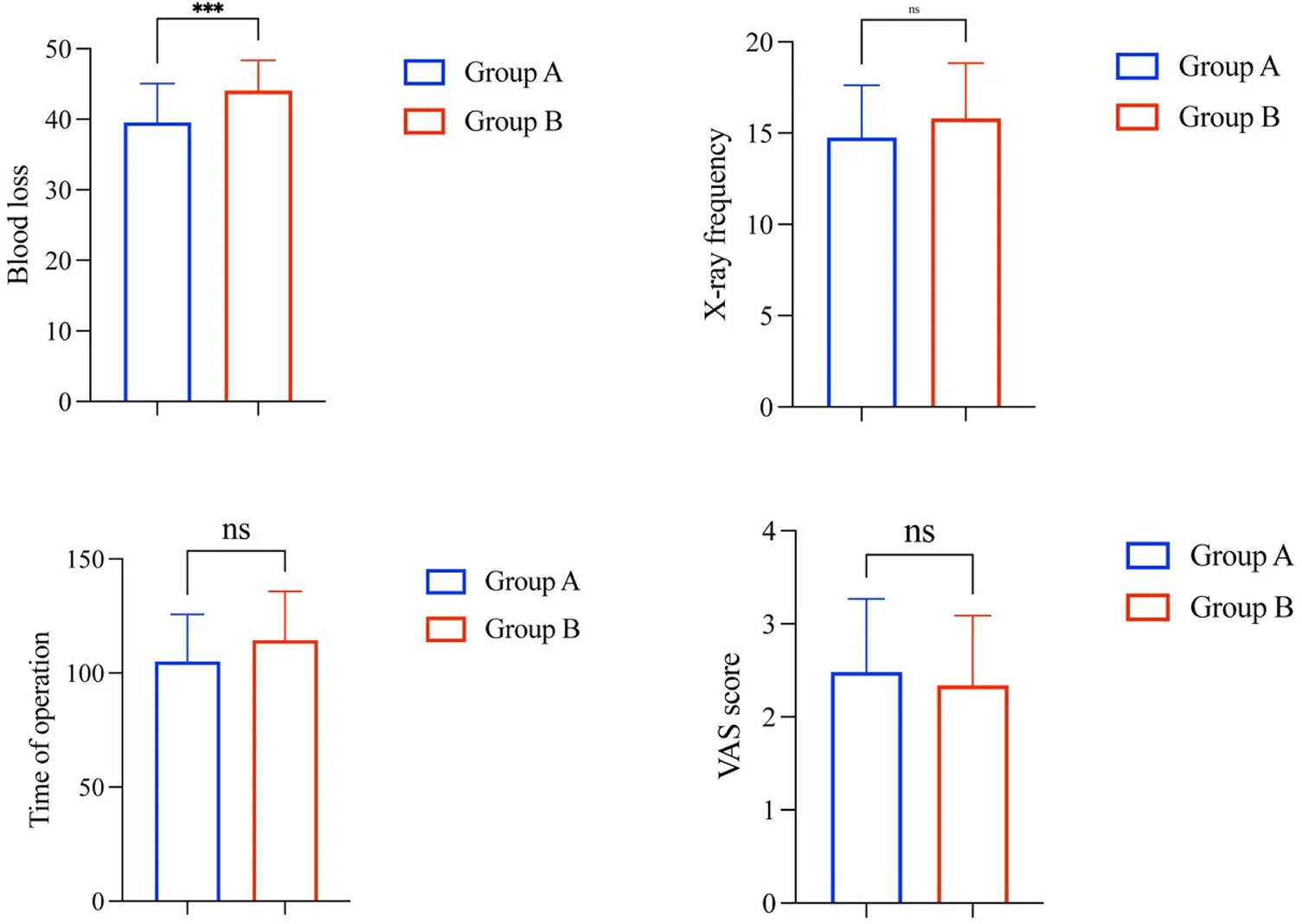
Comparison of perioperative outcomes between group A and group B. Comparison of intraoperative blood loss, operative duration, fluoroscopy frequency, and postoperative day 2 Visual Analog Scale (VAS) pain scores between the isolated Femoral Neck System (FNS) fixation group (Group A, *n* = 33) and the FNS combined with recombinant human bone morphogenetic protein-2 (rhBMP-2) implantation group (Group B, *n* = 32). Data are presented as mean ± standard deviation. Statistical comparisons between groups were performed using independent-samples *t*-test or Mann–Whitney *U* test according to data distribution. FNS, Femoral Neck System; rhBMP-2, recombinant human bone morphogenetic protein-2; VAS, Visual Analog Scale.

No significant intergroup differences were identified in operative duration, intraoperative blood loss, fluoroscopy frequency, or postoperative day 2 Visual Analog Scale (VAS) pain scores (*P* > 0.05; Figure 4). These findings indicated that the addition of local rhBMP-2 implantation did not significantly increase perioperative surgical burden.

### Radiographic outcomes

**Figure 5:**
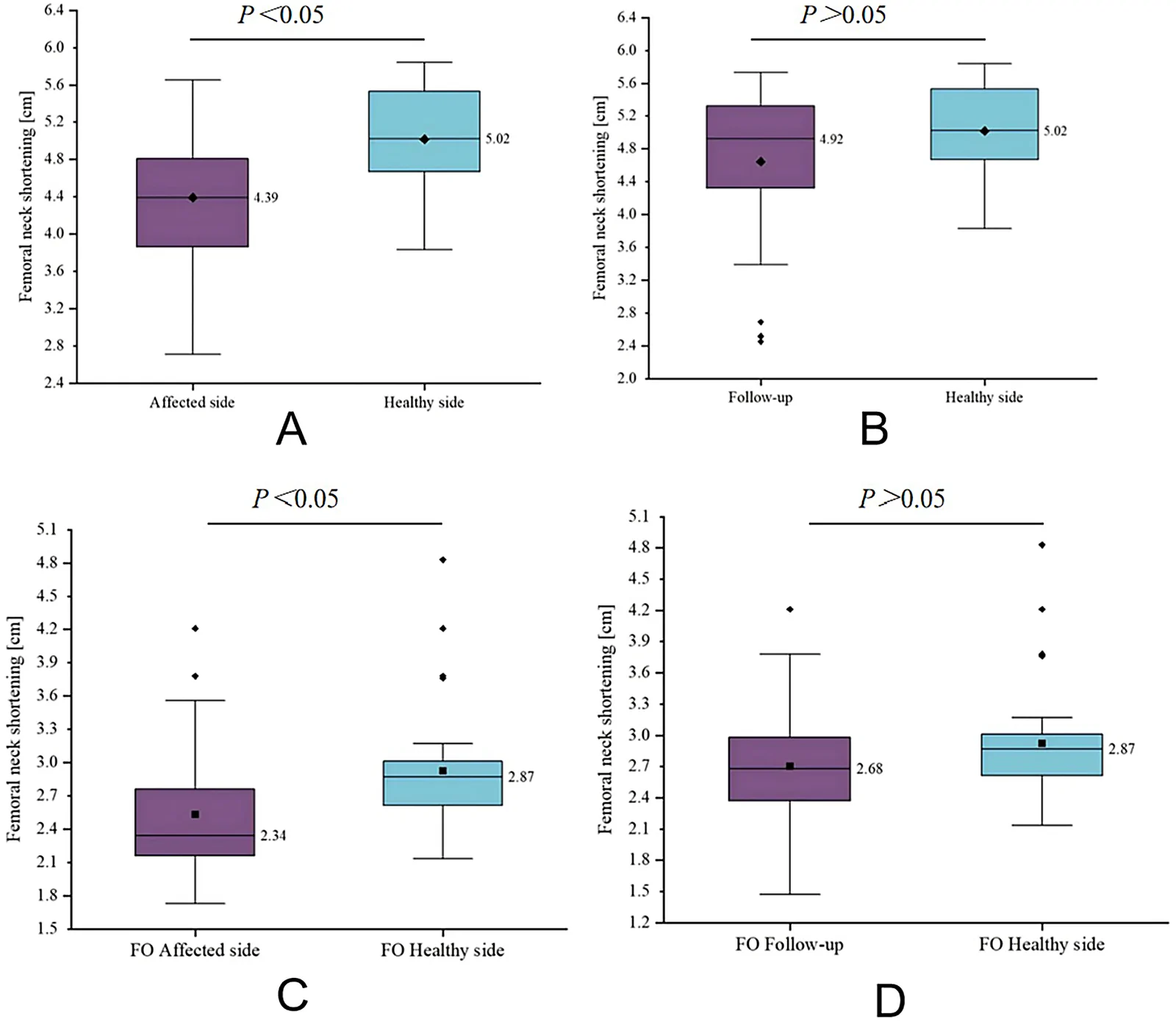
Changes in femoral neck shortening and femoral offset in the isolated FNS fixation group (group A, *n* = 33). **(A)** Significant difference in femoral neck shortening between the affected hip and contralateral healthy hip before surgery (*P* = 0.000). **(B)** No significant difference in femoral neck shortening between the affected hip and contralateral healthy hip at the final follow-up (*P* = 0.061). **(C)** Significant difference in femoral offset between the affected hip and contralateral healthy hip before surgery (*P* = 0.005). **(D)** No significant difference in femoral offset between the affected hip and contralateral healthy hip at the final follow-up (*P* = 0.123). Paired comparisons between affected and contralateral healthy hips were performed using paired-samples *t*-test or Wilcoxon signed-rank test according to data distribution. FNS, Femoral Neck System; FO, femoral offset.

**Figure 6:**
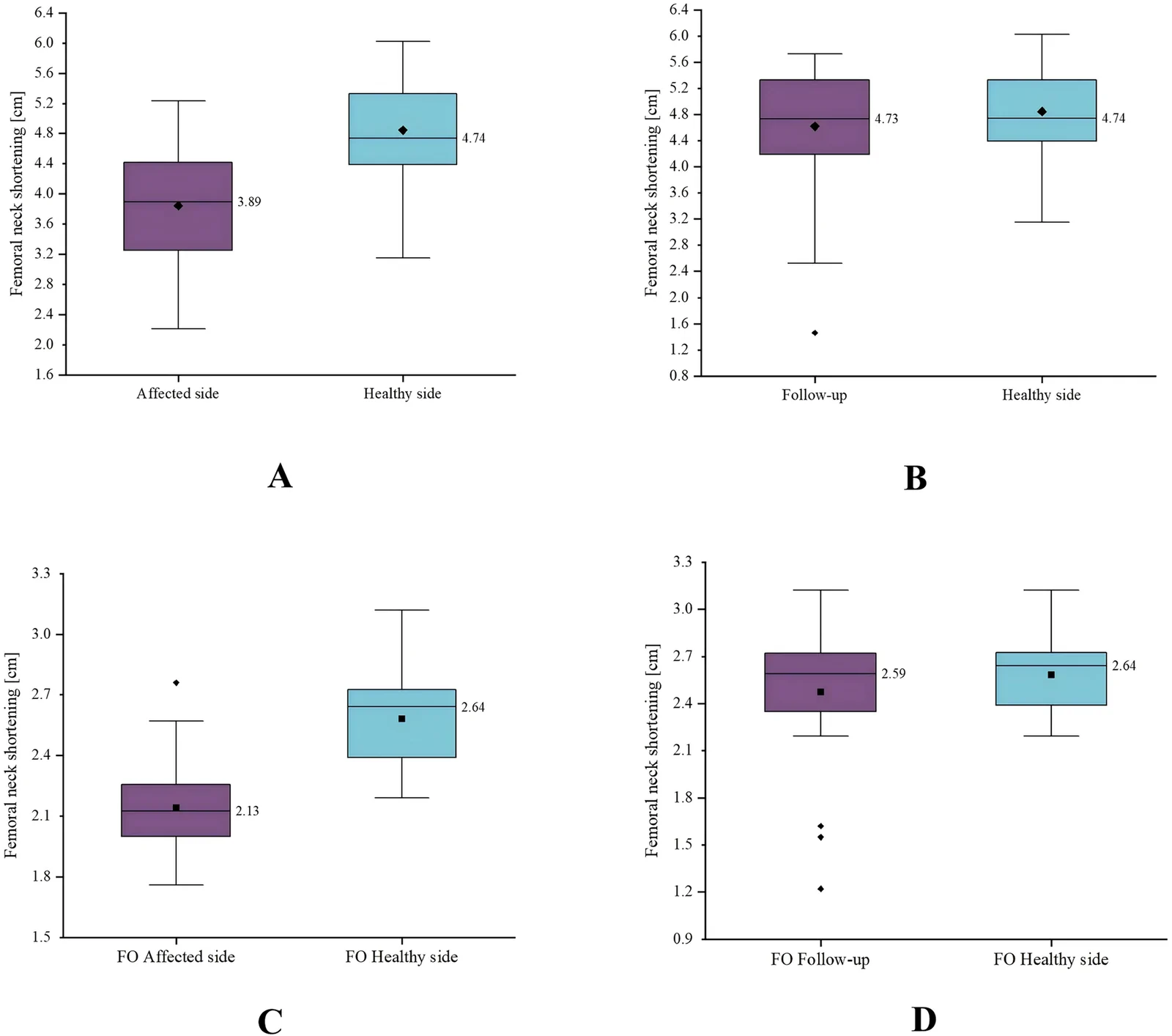
Changes in femoral neck shortening and femoral offset in the FNS combined with rhBMP-2 group (group B, *n* = 32). **(A)** Significant difference in femoral neck shortening between the affected hip and contralateral healthy hip before surgery (*P* = 0.000). **(B)** No significant difference in femoral neck shortening between the affected hip and contralateral healthy hip at the final follow-up (*P* = 0.270). **(C)** Significant difference in femoral offset between the affected hip and contralateral healthy hip before surgery (*P* = 0.000). **(D)** No significant difference in femoral offset between the affected hip and contralateral healthy hip at the final follow-up (*P* = 0.162). Paired comparisons between affected and contralateral healthy hips were performed using paired-samples *t*-test or Wilcoxon signed-rank test according to data distribution. FNS, Femoral Neck System; rhBMP-2, recombinant human bone morphogenetic protein-2; FO, femoral offset.

Statistically significant differences in fracture healing outcomes were observed between the two groups (*P* < 0.05; Table 2). Compared with isolated FNS fixation, the FNS combined with rhBMP-2 group demonstrated improved radiographic healing outcomes. For patients receiving isolated FNS fixation, preoperative femoral neck shortening showed a statistically significant difference compared with the contralateral healthy hip (*P* = 0.000) (Figure 5A). However, no significant bilateral difference was observed at the final follow-up (*P* = 0.061) (Figure 5B). Similarly, preoperative femoral offset demonstrated significant asymmetry between the injured and uninjured sides (*P* = 0.005) (Figure 5C), whereas no statistically significant difference remained at the final follow-up (*P* = 0.123) (Figure 5D). In the FNS combined rhBMP-2 group, preoperative femoral neck shortening was significantly different from the contralateral healthy hip (*P* = 0.000) (Figure 6A), while this difference was no longer significant at the final follow-up (*P* = 0.270) (Figure 6B). Preoperative femoral offset also demonstrated significant bilateral asymmetry (*P* = 0.000) (Figure 6C), with no significant difference observed between the two hips at the final follow-up (*P* = 0.162) (Figure 6D).

**Table 2:** Comparison of postoperative recovery and postoperative complications between the two groups of patients.

Outcome	Group A: FNS alone (*n* = 33)	Group B: FNS combined with rhBMP-2 (*n* = 32)	Statistic value(t/χ^2^)	*P* value
Complete weight-bearing time (weeks)	14.18 ± 1.33	13.00 ± 2.08	2.736	0.008
Fracture healing time (months)	3.69 ± 0.49	3.28 ± 0.46	3.351	0.001
Femoral neck shortening [*n* (%)]	3 (9.1)	2 (6.3)	0.185	0.667
Fracture nonunion [*n* (%)]	2 (6.1)	1 (3.1)	0.318	0.573
Femoral head necrosis [*n* (%)]	6 (18.2)	1 (3.1)	4.223	0.040
Withdrawal of nails [*n* (%)]	0	0	—	—

### Functional outcomes

**Figure 7:**
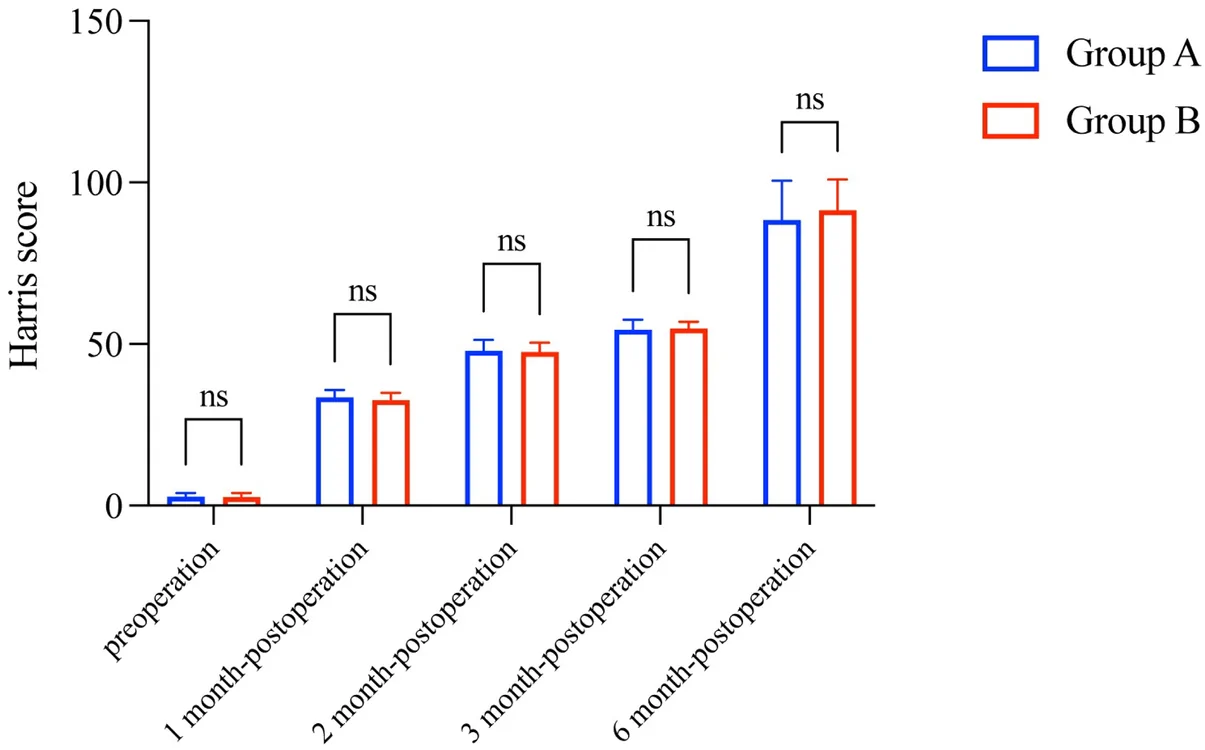
Comparison of postoperative hip function between group A and group B. Comparison of Harris Hip Scores at the final follow-up between the isolated FNS fixation group (Group A, *n* = 33) and the FNS combined with rhBMP-2 implantation group (Group B, *n* = 32). No statistically significant difference was observed between the two groups (*P* > 0.05). Data are presented as mean ± standard deviation. Statistical analysis was performed using an independent-samples *t*-test. FNS, Femoral Neck System; rhBMP-2, recombinant human bone morphogenetic protein-2.

Both groups achieved favorable hip functional recovery after surgery. No statistically significant difference in Harris Hip Scores was observed between Group A and Group B at the final follow-up (*P* > 0.05; Figure 7).

### Psychological outcomes

**Figure 8:**
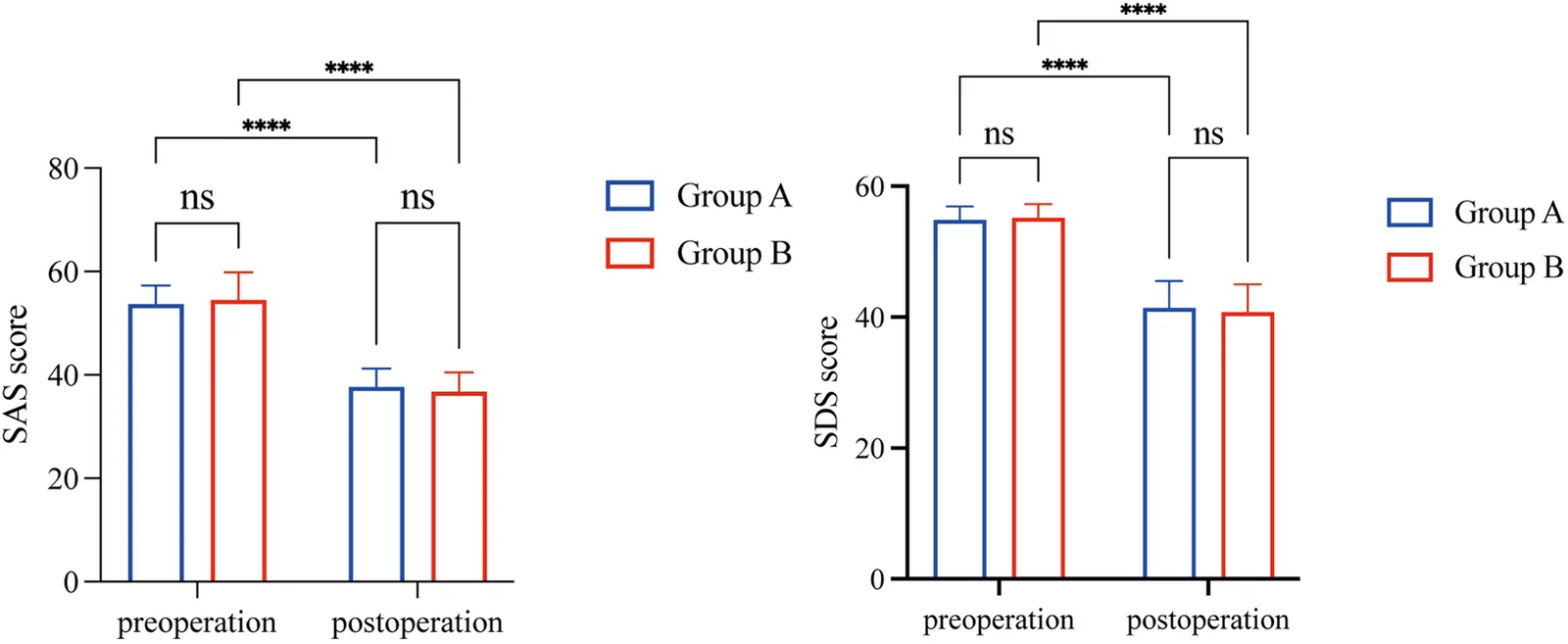
Comparison of psychological outcomes before and after surgery between group A and group B. Comparison of preoperative and postoperative Self-Rating Depression Scale (SDS) and Self-Rating Anxiety Scale (SAS) scores in the isolated FNS fixation group (Group A, *n* = 33) and the FNS combined with rhBMP-2 implantation group (Group B, *n* = 32). Both groups demonstrated significant postoperative reductions in SDS and SAS scores compared with preoperative values (*P* = 0.000). Data are presented as mean ± standard deviation. Intragroup comparisons between preoperative and postoperative values were performed using paired-samples *t*-test. Intergroup comparisons were performed using independent-samples *t*-test. SDS, Self-Rating Depression Scale; SAS, Self-Rating Anxiety Scale; FNS, Femoral Neck System; rhBMP-2, recombinant human bone morphogenetic protein-2.

No statistically significant intergroup differences were observed in preoperative or postoperative SDS and SAS scores between Group A and Group B (*P* > 0.05). However, intragroup analyses demonstrated significant reductions in both SDS and SAS scores from baseline to the final follow-up in Group A (*P* = 0.000). Similarly, Group B showed significant postoperative improvements in SDS and SAS scores compared with preoperative values (*P* = 0.000; Figure 8).

### Complications

**Figure 9:**
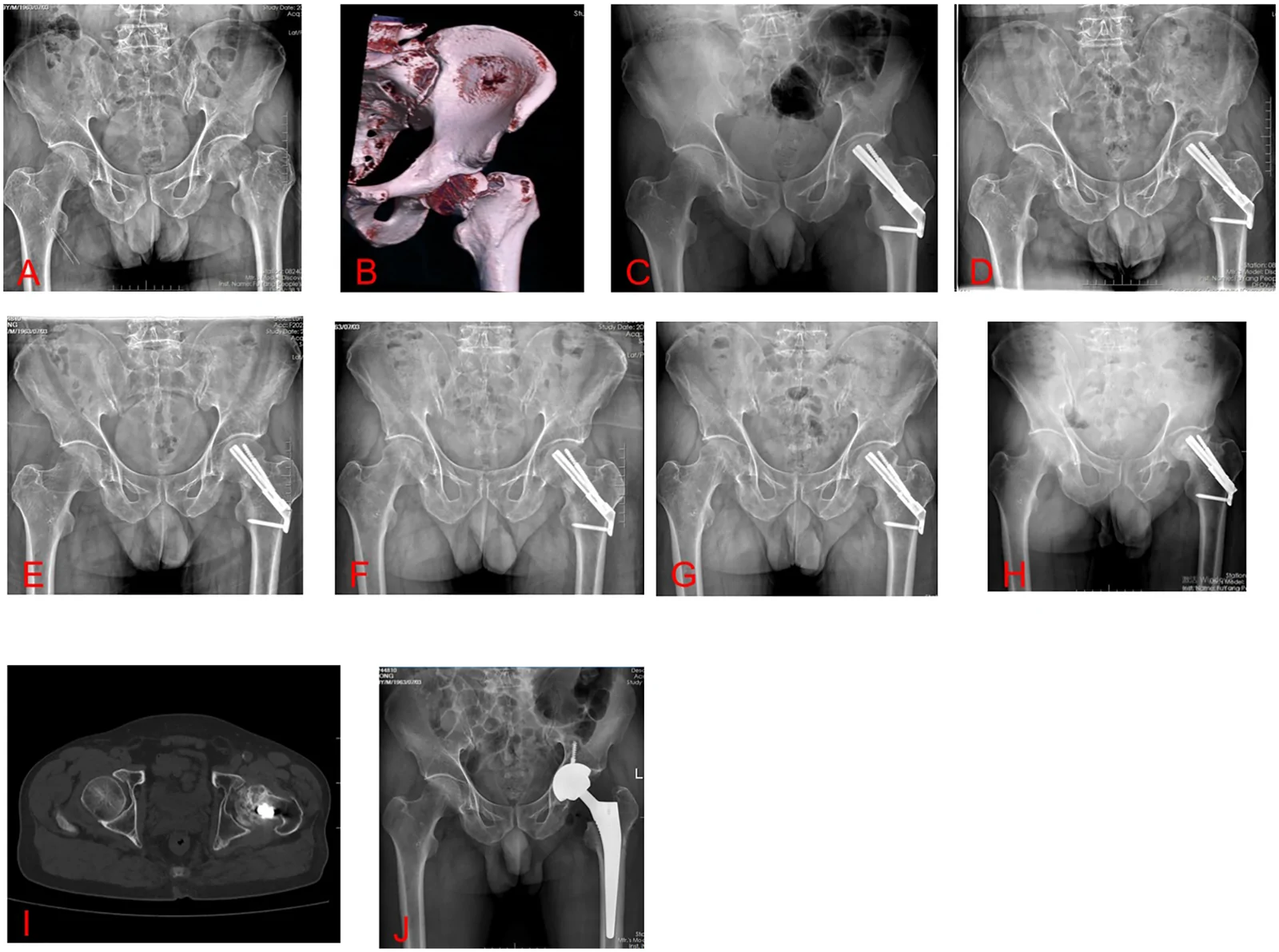
Representative case of femoral head necrosis after isolated FNS fixation. A 52-year-old male patient with a left Garden type III femoral neck fracture underwent internal fixation using the Femoral Neck System. Preoperative anteroposterior radiograph **(A)** and three-dimensional CT reconstruction **(B)** Immediate postoperative anteroposterior radiograph after FNS fixation **(C)** Follow-up radiographs at 1, 2, 3, and 12 months after surgery **(D–G)**. Radiograph demonstrating femoral head necrosis at 16 months postoperatively **(H)**, CT evaluation **(I)**, and subsequent conversion to total hip arthroplasty **(J)**. CT, computed tomography; FNS, Femoral Neck System; Garden classification, Garden fracture classification.

In Group A, six patients developed avascular necrosis of the femoral head, three patients experienced femoral neck shortening, and two patients developed fracture nonunion. Statistically significant differences in fracture healing-related complications were observed between the two groups (*P* < 0.05; Table 2). A representative case involved a 52-year-old male patient diagnosed with a left Garden type III femoral neck fracture who underwent isolated FNS fixation. The patient developed avascular necrosis of the femoral head 16 months after surgery and ultimately underwent conversion to total hip arthroplasty (Figures 9A–J).

### Discussion

Femoral neck fractures in young adults remain a challenging clinical problem because of the high risk of postoperative complications, particularly fracture nonunion and avascular necrosis of the femoral head. High-energy trauma represents the primary injury mechanism in this population, often resulting in complex fracture patterns, compromised vascular supply, and mechanical instability. According to previous clinical criteria, young adult femoral neck fractures generally refer to fractures occurring in patients aged 18–60 years (2, 5, 6). Considering the characteristics of our single-center cohort, patients aged 18–55 years were included in the present study. Achieving anatomical reduction and stable fixation remains the fundamental principle for successful treatment.

Although various fixation techniques, including cannulated compression screws, dynamic hip screws, and the Femoral Neck System (FNS), have been developed for femoral neck fractures (7–16), postoperative complications such as nonunion, femoral neck shortening, and femoral head necrosis remain important clinical concerns. Previous studies have demonstrated that different fixation methods provide distinct biomechanical advantages; however, mechanical stability alone may not completely overcome the limited biological healing potential associated with femoral neck fractures. Femoral head necrosis remains one of the most serious complications after femoral neck fracture fixation, with reported incidence rates ranging from 10% to 30% (17). Therefore, treatment strategies combining mechanical stability with biological enhancement may provide additional clinical benefits.

The FNS was developed to provide stable fixation through a minimally invasive approach and has demonstrated favorable biomechanical characteristics, including improved anti-rotation stability and resistance to displacement, compared with traditional fixation methods. In the present study, both groups achieved satisfactory fixation outcomes, and no implant-related complications such as screw back-out were observed. However, compared with isolated FNS fixation, FNS combined with rhBMP-2 implantation resulted in shorter fracture healing time and lower postoperative complication rates. These findings suggest that biological augmentation may provide additional benefits when combined with stable mechanical fixation in unstable femoral neck fractures.

The potential mechanism underlying the beneficial effects of rhBMP-2 may be related to its osteoinductive properties. rhBMP-2 promotes osteogenic differentiation and bone regeneration through regulation of osteoblast-related biological processes (18–21). In addition to biological factors, postoperative recovery after femoral neck fracture fixation is influenced by multiple clinical and psychological factors. Previous studies have demonstrated that psychological status, including anxiety and depressive symptoms, may affect rehabilitation progress and functional recovery after orthopedic trauma (22, 23). In the present study, both groups showed significant reductions in SDS and SAS scores after treatment, indicating improved psychological status after surgery and rehabilitation. However, no significant differences were observed between groups, suggesting that psychological recovery may be influenced by multiple factors, including rehabilitation process, social support, and individual adaptation (24, 25).

Femoral neck shortening is another important factor affecting postoperative outcomes. Excessive shortening may alter hip biomechanics by reducing femoral offset and affecting abductor muscle function (26). Therefore, maintaining fracture stability and promoting biological healing are essential for preventing structural collapse after fixation. Previous studies have demonstrated that sustained-release rhBMP-2 delivery systems can enhance osteogenic activity and improve bone repair outcomes. Xiong et al. reported that sustained-release rhBMP-2 preparations improved osteogenesis and bone mechanical properties (27). Pan et al. further demonstrated that rhBMP-2/PLGA sustained-release microspheres promoted osteogenesis and vascular regeneration in femoral head necrosis models (28). These findings provide a biological rationale for the potential benefits of rhBMP-2 augmentation observed in our study. Previous studies have also suggested that fracture stability, reduction quality, implant selection, and bone conditions may influence postoperative shortening and fixation failure (29, 30). The FNS provides stable fixation and favorable biomechanical characteristics, which may contribute to maintaining fracture stability during the healing process (31, 32). In the present study, femoral neck shortening occurred in both groups, but the incidence was lower in patients receiving rhBMP-2 augmentation, suggesting that enhanced biological healing may contribute to improved structural maintenance.

The Harris Hip Score is widely used to evaluate functional recovery after femoral neck fracture fixation. In this study, although the FNS combined with rhBMP-2 group demonstrated improved radiographic healing outcomes, no significant difference in Harris Hip Scores was observed between groups. This finding suggests that rhBMP-2 augmentation may primarily influence fracture healing rather than short-term functional recovery. Functional outcomes after femoral neck fracture fixation are also affected by multiple factors, including fracture pattern, reduction quality, rehabilitation protocols, and patient-related factors.

Previous studies have emphasized the importance of early mobilization and standardized rehabilitation following stable fixation of femoral neck fractures. Enhanced recovery strategies may contribute to improved postoperative recovery and reduced complications (33, 34). Therefore, the combination of stable fixation using FNS, biological augmentation with rhBMP-2, and appropriate rehabilitation protocols may represent a promising treatment strategy for unstable femoral neck fractures.

This study has several strengths. First, it evaluated a novel treatment strategy combining mechanical stabilization with biological augmentation in patients with unstable femoral neck fractures. Second, both radiographic outcomes and psychological outcomes were assessed, providing a more comprehensive evaluation of postoperative recovery.

Several limitations should be acknowledged. First, this retrospective single-center study included a relatively small sample size, partly because the clinical application of FNS combined with rhBMP-2 remains relatively limited. Therefore, the findings should be interpreted cautiously, and multicenter prospective studies with larger sample sizes are required. Second, this study only compared isolated FNS fixation with FNS combined with rhBMP-2 implantation. Comparisons with other commonly used fixation methods, such as cannulated compression screws or dynamic hip screws, may provide additional evidence regarding the optimal treatment strategy. Third, potential confounding factors, including fracture severity, reduction quality, comorbidities, and surgeon preference, could not be completely controlled because of the retrospective design. In addition, cost-effectiveness, quality of life, and validated patient-reported outcome measures were not evaluated. Finally, psychological assessment was performed using standardized scales rather than individualized psychological intervention protocols. Future studies incorporating personalized psychological management and longer-term follow-up are warranted.

## Conclusions

In patients aged 18–55 years with unstable femoral neck fractures, FNS combined with local rhBMP-2 implantation was associated with shorter fracture healing time, fewer postoperative complications, and improved radiographic outcomes compared with FNS fixation alone. However, no significant differences were observed in hip functional recovery or psychological outcomes between the two groups. These findings suggest that rhBMP-2 augmentation may provide additional biological benefits when combined with stable mechanical fixation using FNS, although further prospective multicenter studies with larger sample sizes and longer follow-up are required to confirm its clinical efficacy.

## Data Availability

The raw data supporting the conclusions of this article will be made available by the authors, without undue reservation.
